# Cartographic analysis of woodlice fauna of the former USSR

**DOI:** 10.3897/zookeys.176.2372

**Published:** 2012-03-20

**Authors:** Daria M. Kuznetsova, Konstantin B. Gongalsky

**Affiliations:** 1 A.N. Severtsov Institute of Ecology and Evolution, Russian Academy of Sciences, Moscow, Russia

**Keywords:** Woodlice, mean annual airtemperature, database, Russia

## Abstract

An inventory of the woodlice fauna of the former USSR yielded 190 species, 64 of them were recorded from the territory of Russia. According to the cartographic analysis, the limits of distribution of epigean terrestrial isopods over the area, excluding mountains, is explained by temperature. No woodlice records were found outside the isocline of 120 days a year with the mean daily air temperature >10°C. The highest species diversity was found between the isoclines of 180 and 210 days. These areas correspond to forest-steppe and steppe zones.

## Introduction

Studies of spatial differentiation of various taxa are among the most important frontiers of modern biogeography. For some well-studied groups, mainly, vertebrates and plants, such trends are already discovered (Loiselle et al. 2003; Guisan and Thuiller 2005; Grenouillet et al. 2011), but for soil-dwelling invertebrates they are only at the stage of species inventory. However, there are certain groups of invertebrates for which analysis of spatial differentiation is already possible due to the large number of records from different geographical localities. Woodlice are among such groups.

There is no faunistic list of terrestrial isopods for the territory of the former USSR until now, as well as of the territory of Russia. However, there are extensive regional lists (Borutzky 1948, 1953; Zalesskaya and Rybalov 1982; Khisametdinova 2007; Gongalsky and Kuznetsova 2011), and numerous records scattered in the literature devoted to soil macrofauna. At the same time, there are only a few ecological studies about factors affecting woodlice distribution over regions of the former USSR (Gongalsky et al. 2005; Khisametdinova 2009).

The aim of the study is to determine the factors affecting woodlice distribution over the plain area of the former Soviet Union. To achieve this, an inventory of species distribution across the study area was made. The task was to create a database indicating locations with woodlice presence/absence overlaid with several environmental variables values distribution.

## Material and methods

### Database

The first step was to compile a list of species for the study area. We made a database of isopod presence or absence in the locations across the whole territory of the former USSR (both plains and mountains). For each record the database includes information about date, data source, geographical coordinates, location, isopod species list or information about woodlice absence in the soil fauna list, biotope, and natural zone.

Three types of information sources of terrestrial isopod locations were used: i) available literature on soil fauna surveys; ii) collections of the Zoological Museum of Moscow State University (Moscow, Russia) and the Zoological Institute of the Russian Academy of Sciences (St.-Petersburg, Russia); and iii) authors’ personal collections. Here we provide a list of woodlice from the territory of the former USSR since some species and localities were not included in the list of Schmalfuss (2003), although it covered the majority of species. To work with regional databases, a specific list would be useful. Since such a list for this area did not exist, the proposed compilation would be a start to be completed in the future. We used the taxonomic system proposed by Schmalfuss (2003) for species naming. Isopod absence was recorded only in extensively surveyed locations.

For cartographic analysis, 259 locations were chosen, 44 of which with woodlice absence. Due to the difficulty of tracing ecological trends in the mountains, only plain territories were involved into the analysis. Some species were excluded from the analysis: i) synanthropic species and ii) species inhabiting azonal locations, such as sea coasts, caves and anthills.

Then database records with isopod presence or absence locations were laid on the geographic maps to perform cartographic analysis.

### Cartographic analysis

The map of woodlice distribution was visually compared with the maps of environmental factors (mean annual temperature; the period with temperature above 10°C; mean precipitation; permafrost distribution; soil pH and soil type; vegetation type; natural zones) found in the Agricultural Atlas of the USSR (Tulupnikov 1960) and the Geographical Atlas of the USSR (Kolosova 1980). The data were verified using the WorldClim database (Hijmans et al. 2005).

The database is maintained in MS Excel. Cartographic analysis is done in MapInfo 8.5.

## Results and discussion

### Limits of isopod distribution

**Figure 1.**
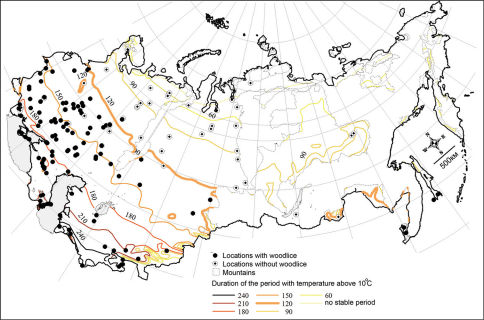
Map of woodlice presence or absence over the plain territory of the former USSR. The duration of period with temperature >10°C is adapted from Geographical Atlas of the USSR (Kolosova 1980).

Woodlice have not been recorded northwards the isocline of 120 days a year with temperature >10°C (Fig. 1). The northern border of woodlice distribution matches the distribution of this parameter. Other parameters did not coincide with isopod distribution as well as with this isocline (data not shown).

### Species diversity

In total, 190 species were recorded from the territory of the former USSR (Appendix 1). Among them, 64 were recorded from the territory of Russia. Northernmost natural zone with woodlice records is southern taiga. No woodlice records were in tundra, northern and middle taiga. The species diversity increases southwards, but decreases in the deserts. However, this may be due to the low number of locations extensively studied to reveal local faunas.

Distribution of isopods is known to be limited by natural factors, such as temperature and moisture (Harding and Sutton 1985, Hopkin 1991). In our study, the limiting factor of woodlice distribution towards the north turned out to be the length of the warm period, expressed as number of days when the temperature was above 10°C. The highest species diversity was observed between isoclines of 180 and 210 days with temperature >10°C. Colder conditions slow down their physiological processes (Hopkin 1991) and limit their distribution. For a better understanding of distribution of woodlice, a Species Distribution Modeling (Elith and Leathwick 2009, Franklin 2009) should be applied, which is a next step in the analysis of the database of Russian isopods.

## Figures and Tables

**Table:** List of woodlice species from the territory of the former USSR. Abbreviations: Ab – Abkhazia, Ar – Armenia, Az – Azerbaijan, Bl – Belarus, Ge – Georgia, Kz – Kazakhstan, Kg – Kyrgyzstan, Lt – Lithuania, Md – Moldova, Ru – Russia, Td – Tajikistan, Tu – Turkmenistan, Ua – Ukraine, Uz – Uzbekistan; S, N, W, E – south, north, west, east. References to authorships of the species can be found in Schmalfuss and Wolf-Schwenninger (2002).

1	*Acaeroplastes kosswigi* Verhoeff, 1941	Az: Nabran’
2	*Agabiformius orientalis* (Dollfus, 1905)	Ru: Volgograd region
3	*Armadillidium azerbaidzhanum* Schmalfuss, 1990	Ar: Khastarak; Az: Baku, Adjikend, Drmbon, Lenkoran, Salyany, Sheki; Ge: Kakhetia, Vashlovan; Ru: Rostov, Stavropol regions, N Osetia
4	*Armadillidium granulatum* Brandt, 1833	Ru: Krasnodar region; Ua: S Crimea
5	*Armadillidium nasatum* Budde-Lund, 1885	Ru: Moscow; Ab: Sukhum
6	*Armadillidium opacum* (C.Koch, 1841)	Ua: Kiev
7	*Armadillidium pallasii* Brandt, 1833	Ab: Sukhum; Ua: Crimea, Odessa; Ru: Krasnodar region
8	*Armadillidium pictum* Brandt, 1833	Ua: Crimea
9	*Armadillidium pulchellum* (Zenker, 1798)	Lt: Vilnius
10	*Armadillidium traiani* Demianowicz, 1932	Md
11	*Armadillidium versicolor* Stein, 1859	Ru: Penza, Saratov, Tula regions
12	*Armadillidium vulgare* Latreille, 1804	Ab: Sukhum; Az; Ge: Adygeni, Tbilisi; Ru: Dagestan, Krasnodar, Kaluga, Volgograd, Rostov regions; Ua: Crimea, Kiev, Odessa
13	*Armadillidium zenckeri* Brandt, 1833	Ua: Crimea, Zakaspyisk region
14	*Armadillo alievi* Schmalfuss, 1990	Az: Baku, Kobustan
15	*Armadillo officinalis* Dumeril, 1816	Ru: Krasnodar region; Ua: S Crimea, Odessa
16	*Armadilloniscus ellipticus* (Harger, 1878)	Ru: Krasnodar region; Ab: Gagry
17	*Borutzkyella revasi* (Borutzky, 1973)	Ab: Gudauty region
18	*Buddelundiella cataractae* Verhoeff, 1930	Ge: Tskhaltubo
19	*Caucasocyphonethes cavaticus* Borutzky, 1948	Ru: Krasnodar region
20	*Caucasoligidium cavernicola* Borutzky, 1950	Ab: Gudauty, Sukhum; Ge: Gogolety
21	*Caucasonethes borutzkyi* Verhoeff, 1932	Ge: Tskhaltubo
22	*Chaetophiloscia cellaria* Dollfus, 1884	Ru: Rostov region
23	*Chaetophiloscia elongata* Dollfus, 1884	Ua: Crimea
24	*Chaetophiloscia hastata* Verhoeff, 1929	Ru: Krasnodar region
25	*Colchidoniscus kutaissianus* Borutzky, 1974	Ge: Tskhaltubo
26	*Cylisticoides angulatus* Schmalfuss, 2003	Az: Istisu, Lenkoran, Yardymly; Kz: Astana; Ru: Krasnodar region
27	*Cylisticus albomaculatus* Borutzky, 1957	Ru: Rostov, Voronezh, Volgograd regions
28	*Cylisticus arnoldii* Borutzky, 1961	Ua: Kharkov, Zmiev
29	*Cylisticus birsteini* Borutzky, 1961	Ru: Krasnodar region
30	*Cylisticus caucasius* Verhoeff, 1917	Ab: Gudauty, Gagry, Kelassuri, Sukhum; Ge: Kutaisi, Tkibuli, Tskhaltubo, Shovi; Ru: Krasnodar region
31	*Cylisticus convexus* De Geer, 1778	Ru: Chelyabinsk, Kaluga, Moscow, Rostov, Voronezh regions, Ua: S Crimea, Kiev
32	*Cylisticus cretaceus* Borutzky, 1957	Ru: Rostov region; Ua: Lugansk region
33	*Cylisticus desertorum* Borutzky, 1957	Ru: Rostov, Stavropol regions; Ua
34	*Cylisticus giljarovi* Borutzky, 1977	Ru: Adygea, Stavropol, Krasnodar regions, N Osetia
35	*Cylisticus iners* Budde-Lund, 1880	Ar: Azizbekov, Leninakan, Tshakhkavan; Az: Airidja, Mardakert, Zakatalinsk regions; Ru: Chechnya; Ge: Manglisi, Shuahevi
36	*Cylisticus lencoranensis* Borutzky, 1977	Az: Prishib
37	*Cylisticus mitis* Budde-Lund, 1885	Ge: Kutaisi
38	*Cylisticus orientalis* Borutzky, 1939	Ru: Orenburg region
39	*Cylisticus rotabilis* Budde-Lund, 1885	Ua: S Crimea
40	*Cylisticus sarmaticus* Borutzky, 1977	Ru: Rostov region; Ua: Zaporozhye region
41	*Cylisticus silvestris* Borutzky, 1957	Ru: Moscow, Rostov, Stavropol regions
42	*Cylisticus strouhali* Borutzky, 1977	Ar: W Vanadzor, Spitaki
43	*Desertoniscus birsteini* Borutzky, 1945	Tu
44	*Desertoniscus bulbifrons* Borutzky, 1945	Tu
45	*Desertoniscus elongatus* Borutzky, 1945	Tu
46	*Desertoniscus kirghizicus* Borutzky, 1978	Kg
47	*Desertoniscus reductus* Borutzky, 1978	Td
48	*Desertoniscus subterraneus* Verhoeff, 1930	Kg; Tu: (Kizil-arvat); Td: Samgar massif
49	*Desertoniscus tekinus* Borutzky, 1945	Tu
50	*Desertoniscus zhelochovtzevi* Borutzky, 1945	Uz
51	*Detonella papillicornis* (Richardson, 1904)	Ru: Kamchatka, Sakhalin regions
52	*Halophiloscia couchii* (Kinahan, 1858)	Ru: Krasnodar region; Ua: Crimea
53	*Haplophthalmus danicus* Budde-Lund, 1880	Ru: Krasnodar, Rostov regions
54	*Hemilepistoides messerianus* Borutzky, 1945	Tu
55	*Hemilepistus buddelundi* Borutzky, 1945	Tu
56	*Hemilepistus communis* Borutzky, 1945	Td: Samgar massif
57	*Hemilepistus crenulatus* (Pallas, 1771)	Td: Samgar massif; Tu: Central Karakum, Kyzyl-Arvat; Uz: Zakaspiysk, Fergana regions; Kg
58	*Hemilepistus cristatus* Budde-Lund, 1885	Tu: Kyzyl-Arvat
59	*Hemilepistus elongatus* Budde-Lund, 1885	Ru: Rostov, Stavropol regions; Tu: SW part
60	*Hemilepistus fedtschenkoi* (Uljanin, 1875)	Kz: Semipalatinsk;Tu: Krasnovodsk; Uz: Bukhara, Samarkand, Syrdaryinsk, Zakaspyisk regions
61	*Hemilepistus heptneri* Borutzky, 1945	Tu
62	*Hemilepistus klugii* (Brandt, 1833)	Az: Baku
63	*Hemilepistus magnus* Borutzky, 1945	Uz
64	*Hemilepistus nodosus* Budde-Lund, 1885	Tu; Kz
65	*Hemilepistus pavlovskii* Borutzky, 1954	Kz
66	*Hemilepistus reductus* Borutzky, 1945	Uz: Bukhara, Samarkand, Syrdaryinsk regions
67	*Hemilepistus rhinoceros* Borutzky, 1958	Kz
68	*Hemilepistus ruderalis* (Pallas, 1771)	Ru: Volgograd region; Kz: Djanybek
69	*Hemilepistus russonovae* Borutzky, 1951	Az: Baku
70	*Hemilepistus zachvatkini* Verhoeff, 1930	Td: Samgar massif
71	*Hyloniscus riparius* C. Koch, 1838	Ru: Moscow, Penza, Pskov, Rostov, Tula regions; Ua: Kiev region
72	*Leptotrichus panzerii* (Audonin, 1826)	Ua: Crimea
73	*Leptotrichus tauricus* Budde-Lund, 1885	Ua: Crimea
74	*Ligia cinerascens* Budde-Lund, 1885	Ru: Kurily islands
75	*Ligia italica* Fabricius, 1798	Ua: Crimea
76	*Ligia pallasii* Brandt, 1833	Kadakh?
77	*Ligidium birsteini* Borutzky, 1950	Ab: Gagry
78	*Ligidium cavaticum* Borutzky, 1950	Ru: Krasnodar region
79	*Ligidium fragile* Budde-Lund, 1885	Ab: Sukhum
80	*Ligidium germanicum* Verhoeff, 1901	Md
81	*Ligidium hypnorum* Cuvier, 1792	Ab: Sukhum; Bl: Belovezha National Park; Ru: Tver, Kaluga, Moscow regions; Ua: Crimea, Kiev
82	*Ligidium margaritae* Borutzky, 1955	Kz: Alma-Ata
83	*Ligidium nodulosum* Verhoeff, 1918	Ab: Gagry
84	*Ligidium shadini* Borutzky, 1948	Td
85	*Ligidium tauricum* Verhoeff, 1930	Ua: Crimea
86	*Ligidium zaitzevi* Borutzky, 1950	Ab: Sukhum
87	*Ligidium zernovi* Borutzky, 1948	Kg
88	*Mingrelloniscus inchhuricus* Borutzky, 1974	Ge: Megrelia
89	*Nagurus matekini* Borutzky, 1959	Kg
90	*Oniscus asellus* Linne, 1758	Lt: Vilnius; Ru: Pskov region; Ua: Kiev
91	*Parcylisticus armenicus* Borutzky, 1970	Ar: Daralagez
92	*Parcylisticus dentifrons* Budde-Lund, 1885	Az: Kutkashen; Ge: Manglisi; Ru: Astrakhan, Stavropol regions, Chechnya, Dagestan, Kabardino-Balkaria, N Osetia; Ua: Crimea
93	*Parcylisticus georgianus* Schmalfuss, 2003	Ge: Adigeni, Batumi, Kutaisi, Mestia
94	*Parcylisticus golovatchi* Schmalfuss, 2003	Az: Shikahokh
95	*Parcylisticus mrovdaghicus* (Borutzky, 1970)	Az: Avash, Dashsalty, Kelbadjar, Kirovobad, Lenkoran, Zuvand
96	*Parcylisticus urartuensis* Borutzky, 1970	Ar
97	*Parcylisticus zangezuricus* Borutzky, 1970	Ar
98	*Platyarthrus armenicus* Borutzky, 1976	Ar: Megri
99	*Platyarthrus hoffmannseggii* Brandt, 1833	Ru: Krasnodar region
100	*Platyarthrus luppovae* Borutzky, 1953	Td
101	*Platyarthrus mesasiaticus* Borutzky, 1976	Tu
102	*Platyarthrus ocellatus* Borutzky, 1953	Td
103	*Platyarthrus schoblii* Budde-Lund, 1885	Ua: Crimea
104	*Porcellio bistriatus* Budde-Lund, 1885	Ab: Sukhum; Ru: Krasnodar region
105	*Porcellio crassicornis* C. Koch, 1841	Bl: Minsk
106	*Porcellio dilatatus* Brandt, 1833	Ar: Sevan
107	*Porcellio laevis* Latreille, 1804	Ab: Sukhum; Ru: Altay, Kalmykia, Moscow, Primorie, Rostov regions; Ua: S Crimea, Odessa; Uz: Bukhara region
108	*Porcellio lamellatus* Budde-Lund, 1885	Ua: Crimea
109	*Porcellio obsoletus* Budde-Lund, 1885	Ua: S Crimea
110	*Porcellio scaber* Latreille, 1804	Bl: Belovezha, Berezinsky reserves; Lt: Vilnius; Ru: Belgorod, Kaluga, Moscow, Nizhni Novgorod, Primorie, Rostov regions, Kamchatka, S Kuruly islands, Sakhalin; Ua: Kiev, Kremenetz, Vinnickaya region (Yampol’)
111	*Porcellio spinicornis* Say, 1818	Bl: Minsk; Lt: Vilnius; Md; Ru: Kaluga, Leningrad, Moscow Pskov regions; Ua: Kiev
112	*Porcellio uljanini* Budde-Lund, 1885	Ua: Crimea
113	*Porcellio variabilis* Lucas, 1849	Ua: Crimea
114	*Porcellionides approximatus* Budde-Lund, 1885	Md; Ru: Stavropol region; Ua: Crimea
115	*Porcellionides linearis* (Budde-Lund, 1885)	Uz: Nukus
116	*Porcellionides pruinosus* Brandt, 1833	Ab: Sukhum; Ar: Shorzha; Az: Baku, Khachmas, Nabran; Ru: Baikal, Volgograd, Krasnodar region, Moscow, Rostov, Saratov regions; Ua: Crimea
117	*Porcellionides rectifrons* (Budde-Lund, 1885)	Ua: Crimea
118	*Porcellium collicola* (Verhoeff, 1907)	Md
119	*Porcellium conspersum* C. Koch, 1841	Bl: Belovezha Reserve; Ua
120	*Protracheoniscus abricossovi* Borutzky, 1945	Tu
121	*Protracheoniscus alabashensis* Borutzky, 1959	Kg
122	*Protracheoniscus almaatinus* Borutzky, 1975	Kz: Alma-Ata
123	*Protracheoniscus anatolii* Borutzky, 1959	Kg
124	*Protracheoniscus armenicus* Borutzky, 1975	Ge: Megri
125	*Protracheoniscus asiaticus* (Uljanin, 1875)	Ru: Moscow, Nizhni Novgorod, Rostov, Ryazan’ regions; Td: Smagar massif
126	*Protracheoniscus atrecicus* Borutzky, 1945	Tu: Bugdaily
127	*Protracheoniscus bugdajliensis* Borutzky, 1975	Tu: Bugdaily
128	*Protracheoniscus cristatus* Borutzky, 1945	Az: Lenkoran, Sara isl.; Tu
129	*Protracheoniscus darevskii* Borutzky, 1975	Ar: Megri
130	*Protracheoniscus delilensis* Borutzky, 1945	Tu
131	*Protracheoniscus desertorum* Verhoeff, 1930	Turkestan?
132	*Protracheoniscus digitifer* Borutzky, 1945	Tu
133	*Protracheoniscus fossuliger* Verhoff, 1901	Ru: Krasnodar, Rostov regions
134	*Protracheoniscus giljarovi* Borutzky, 1957	Ru: Rostov region; Ua: Lugansk region
135	*Protracheoniscus gissarensis* Borutzky, 1975	Td: Dushanbe
136	*Protracheoniscus hirsutulus* Verhoff, 1930	Uz: Tashkent
137	*Protracheoniscus kopetdagicus* Borutzky, 1945	Tu
138	*Protracheoniscus kryszanovskii* Borutzky, 1957	Ru: Volgograd region, Kalmykia
139	*Protracheoniscus latus* (Uljanin, 1875)	Td: Zeravshvan valley
140	*Protracheoniscus litoralis* (Budde-Lund, 1885)	Ua: Crimea
141	*Protracheoniscus major* (Dollfus, 1903)	Ru: Rostov region; Ua: Kiev
142	*Protracheoniscus maracandicus* (Uljanin, 1875)	Td: Smagar massif; Uz: Bukhara, Samarkand, Syrdaryinsk regions
143	*Protracheoniscus marginatus* (Uljanin, 1875)	Ua: Crimea
144	*Protracheoniscus nogaicus* Demianowitz,1931	Md; Ru: Rostov region
145	*Protracheoniscus orientalis* (Uljanin, 1875)	Az: Baku, Mardakert; Bl: Berezinsky reserve; Kz: Mangyshlak; Tu: Bugdaily; Ru: Moscow, Orenburg, Primorie regions; Ua: Kremenetz, Odessa regions, Uz: Nukus; Zakaspyisk region
146	*Protracheoniscus panphilovi* Borutzky, 1959	Kg
147	*Protracheoniscus politus* (C. Koch, 1841)	Ru: Moscow region
148	*Protracheoniscus scythicus* Demianowicz, 1932	Md
149	*Protracheoniscus steinbergi* Borutzky, 1961	TU: SW part
150	*Protracheoniscus taschkentensis* Verhoeff, 1930	Uz: Tashkent; Td
151	*Protracheoniscus tashausicus* Borutzky, 1976	Tu; Ru: Rostov region
152	*Protracheoniscus topczievi* Borutzky, 1975	Ru: Krasnodar, Rostov regions; Ua: Zaporozhye region
153	*Protracheoniscus tuberculatus* (Borutzky, 1945)	Tu
154	*Protracheoniscus turcomanicus* Borutzky, 1945	Tu
155	*Protracheoniscus tzvetkovi* Borutzky, 1975	Kz: Alma-Ata, Uzun-Agach; Ru: Moscow region?
156	*Protracheoniscus uljanini* Borutzky, 1953	Td
157	*Protracheoniscus verhoeffi* Strouhal, 1929	Ge: Tbilisi
158	*Protracheoniscus zenkevitschi* (Borutzky, 1945)	Tu
159	*Psachonethes czerkessicus* Borutzky, 1969	Ru: Krasnodar region
160	*Pseudobuddelundiella hostensis* Borutzky, 1967	Ru: Krasnodar region
161	*Pseudobuddelundiella ljovuschkini* Borutzky, 1967	Ru: Krasnodar region
162	*Schizidium davidi* (Dollfus, 1887)	Az: Divichi
163	*Schizidium golovatchi* Schmalfuss, 1988	Ar: Shikalyukh; Az: Baku; Ge: Batumi
164	*Schizidium reinoehli* Schmalfuss, 1988	Ru: Rostov region
165	*Tadzhikoniscus coecus* Borutzky, 1976	Td
166	*Tauroligidium stygium* Borutzky, 1950	Ua: Crimea
167	*Tauronethes lebedinskyi* Borutzky, 1949	Ua: Crimea
168	*Titanethes albus* (C. Koch, 1841)	Ua: Crimea
169	*Trachelipus azerbaidzhanus* Schmalfuss, 1986	Az: E part
170	*Trachelipus caucasius* (Verhoeff, 1918)	Ab: Gagry; Ru: Krasnodar region
171	*Trachelipus difficilis* Radu, 1950	Bl: Belovezha, Berezinsky reserves; Ua: S Crimea
172	*Trachelipus ensiculorum* Verhoeff, 1949	Ar: Yerevan
173	*Trachelipus gagriensis* (Verhoeff, 1918)	Ab: Gagry
174	*Trachelipus kervillei* (Arcangeli, 1938)	Ru: Rostov region
175	*Trachelipus lignaui* (Verhoeff, 1918)	Ab: Gagry; Ru: Rostov region
176	*Trachelipus longipennis* Budde-Lund, 1885	Ab; Ua: S Crimea
177	*Trachelipus lutschnikii* (Verhoeff, 1933)	Ru: Krasnodar region
178	*Trachelipus rathkii* Brandt, 1833	Ab: Sukhum; Az: Airidja; Ar: Chaldyr; Ge: Kutaisi; Bl: Belovezha pusha; Lt: Vilnius; Md; Ru: Belgorod, Kursk, Tver, Maryi-El, Kaluga, Mordovia, Moscow, Penza, Rostov, Leningrad, Saratov, Tula regions; Ua: Crimea, Kiev
179	*Trachelipus razzautii* (Arcangeli, 1913)	Ru: Krasnodar region
180	*Trachelipus sarculatus* (Budde-Lund, 1896)	Ua: Crimea
181	*Trachelipus trachealis* Budde-Lund, 1885	Md
182	*Trichoniscus aphonicus* Borutzky, 1977	Ab
183	*Trichoniscus gudauticus* Borutzky, 1977	Ab
184	*Trichoniscus pusillus* Brandt, 1833	Ua: Crimea, Kiev
185	*Trichoniscus pygmaeus* Sars, 1898	Ru: Krasnodar region
186	*Turanoniscus anacanthotermitis* Borutzky, 1969	Uz: Tashkent
187	*Tylos granuliferus* Budde-Lund, 1885	Ru: Primorie region, S Kuril Islands
188	*Tylos ponticus* Grebnicki, 1874	Ua: Crimea, Odessa
189	*Typhloligidium coecum* (Carl, 1904)	Ua: Crimea
190	*Typhloligidium karabijajlae* Borutzky, 1962	Ua: Crimea
